# Association of Coffee, Tea, and Caffeine Consumption With All-Cause Risk and Specific Mortality for Cardiovascular Disease Patients

**DOI:** 10.3389/fnut.2022.842856

**Published:** 2022-06-23

**Authors:** Haotian Zheng, Fan Lin, Ning Xin, Linxin Yang, Pengli Zhu

**Affiliations:** ^1^The Shengli Clinical Medical College, Fujian Medical University, Fuzhou, China; ^2^Department of Geriatric Medicine, Fujian Provincial Hospital, Fuzhou, China

**Keywords:** all-cause mortality, CVD, caffeine, decaffeinated coffee/tea, coffee and tea, specific mortality

## Abstract

**Aim:**

The aim of the study was to examine the relationship between coffee, tea, caffeine consumption and risk of all-cause death and cardiovascular disease (CVD) death in CVD population.

**Methods:**

This cohort study included 626 CVD participants aged ≥18 years old who derived from the National Health and Nutrition Examination Surveys (NHANES) database 2003–2006. The end time of follow-up was 2015, and with a median follow-up time of 113.5 (63, 133) months. CVD death was defined as a death caused by congestive heart failure (CHF), coronary heart disease (CHD), angina pectoris, heart attack or stroke. Cox model and competitive-risk model were used to explore the relationship of coffee, tea, caffeine, decaffeinated coffee/tea on the risk of the all-cause death and CVD death for CVD population, respectively. Additionally, we explored the effect of urinary caffeine and caffeine metabolites on all-cause death.

**Results:**

All patients were divided into survival group (*n* = 304), non-CVD death group (*n* = 223), and CVD death group (*n* = 99). The incidence of all-cause death and CVD death was ~51.44 and 15.81% in the study. After adjusting age, body mass index (BMI), cancer, estimated glomerular filtration rate (eGFR), energy, the history of CVD medications, carbohydrate and family income to poverty ratio (PIR), the results suggested coffee, caffeine, iced tea and hot tea consumption (≥4 cups per day) were associated with an increased risk of the all-cause death in CVD patients; while hot tea (1–3 cups per day), decaffeinated coffee/iced tea/hot tea could reduce the risk of the all-cause death. Likewise, coffee, caffeine, iced tea (≥4 cups per day), hot tea, decaffeinated iced tea/ hot tea (Always) could enhance the risk of the CVD death in CVD population. We also found that 1-methylxanthine showed a significant positive association on the risk of all-cause death in CVD population.

**Conclusion:**

Our study indicated that higher consumption of coffee, tea and caffeine could increase the risk of all-cause and CVD death for CVD patients.

## Introduction

Cardiovascular disease (CVD) has been recognized as a frequently diagnosed chronic disease in the world, and remains the major cause of premature death and disability in human beings (1, 2). Recent evidence suggests that the prevalence rate of CVD was over 500 million people and ~20 million people died from CVD per year (3), which seriously reduced quality of life and increased the burden of disease for many families. Previous studies have been observed that dietary factors might play an important role in the development of CVD mortality (4–6).

It is reported that coffee and tea are the most widely consumed beverages worldwide after water, and also regarded as the principal source of caffeine (7). Traditionally, people have been advised to reduce their coffee, tea and caffeine intake because it may increase some indicators which were harmful to physical health, such as blood pressure, total cholesterol, and triglycerides (8). However, several studies have found that chronic coffee consumption, tea consumption and caffeine intake could reduce the risk of CVD death through anti-inflammatory, anti-oxidant, lower blood sugar and fat functions (9–11), but these studies excluded the patients with CVD in their population selection. To the best of our knowledge, few studies in recent years have focused on the relationship of coffee, tea, caffeine intake and death in patients diagnosed with CVD, and these results remained controversial. de Vreede-Swagemakers et al. reported that heavy coffee consumption was associated with an increased risk of sudden cardiac death in patients who suffered sudden cardiac arrest and had a history of coronary artery disease (12). Conversely, in the study of Ribeiro et al., they pointed out that coffee consumption was related to lower risk of cardiovascular mortality and all-cause mortality in patients with myocardial infarction (13). Also, caffeine intake was thought to reduce cerebral blood flow for patients with ischemic stroke, which brought adverse clinical outcomes for patients (14). For a study of US women with CVD, there was no relationship between long-term consumption of filtered caffeinated coffee and all-cause or CVD death (15).

Herein, for patients with CVD, we conducted a follow-up study to investigate the relationship between coffee consumption, tea consumption, caffeine intake, decaffeinated coffee, decaffeinated tea on the risk of all-cause death and CVD death based on the National Health and Nutrition Examination Surveys (NHANES) database, and explore the effect of caffeine metabolites on all-cause death.

## Methods

### Study Design and Participants

All information of participants in this cohort study were obtained from NHANES database (16), which was a program of studies to evaluate the health and nutritional status of adults and children in the United States. National Center for Health Statistics (NCHS) has administered NHANES, a nationally representative survey consisting of about 5,000 persons from 15 different counties each year, continuously since 1999. The survey combines interviews and physical examinations, including demographic, socioeconomic, dietary, and health-related questions, medical, dental, and physiological measurements and laboratory tests (17) (https://www.cdc.gov/nchs/nhanes/index.htm).

Our study collected data of 20,470 participants between 2003 and 2006 from NHANES database. Included criteria: patients who were aged ≥18 years old and diagnosed as CVD in the present analysis. Simultaneously, patients who met any of the following criteria need to be excluded, (1) participants without CVD; (2) participants with incomplete information of coffee and tea consumption; (3) participants with extreme total energy intakes of <500 or >5,000 kcal/day for women and <500 or >8,000 kcal/day for men. All data of this study came from a publicly available database and ethical approval was obtained from the institutional review board at the NCHS Ethics Review Board (17).

### Data Collection

Baseline data were collected, including age, gender, race, body mass index (BMI), education, family income to poverty ratio (PIR), smoking status, alcohol (gm), cancer, hypertension, diabetes, the history of CVD medications (18), cholesterol, protein (gm), total fat (gm), carbohydrate (gm), estimated glomerular filtration rate (eGFR, mL/min/m^2^), energy (10 kcal), coffee intake (cup/day), caffeine (100 mg), iced tea intake (cup/day), hot tea intake (cup/day), decaffeinated coffee, decaffeinated iced tea, decaffeinated hot tea. The NHANES asked all participants to provide some responses that had beverage consumption over the past 12 months (19); the survey question was “Did you drink coffee?” if your answer was yes, a follow-up question was asked “Did you drink how many cups of coffee, caffeinated or decaffeinated?” response options included <1 cup per day, 1–3 cups per day, ≥4 cups per day; then, they were asked “Did you drink decaffeinated, and how often do you drink decaffeinated coffee?” the choices in this question were, almost never, about a quarter to three quarters of the time, or always. The same question structures that contained frequency, decaffeinated vs. caffeinated types applied to iced tea and hot tea.

In addition, accurately determining caffeine dose from coffee and tea might be challenging (20, 21). Herein, urinary caffeine and caffeine metabolite levels have been proposed as an effective indicator of assessing caffeine intake. All NHANES participants were required to provide urine samples in a mobile examination center (MEC), and used ultra-high performance liquid chromatography-electrospray ionization-tandem quadrupole mass spectrometry to analyze the urine samples for caffeine and caffeine metabolites (21), including 1-methyluric acid (1U), 3-methyluric acid (3U), 7-methyluric acid (7U), 1,3-dimethyluric acid (13U), 1,7-dimethyluric acid (17U), 3,7-dimethyluric acid (37U), 1,3,7-trimethyluric acid (137U), 1-methylxanthine (1X), 3-methylxanthine (3X), 7-methylxanthine (7X), theophylline (1,3-dimethylxanthine, 13X), paraxanthine (1,7-dimethylxanthine, 17X), theobromine (3,7-dimethylxanthine, 37X), caffeine (1,3,7-trimethylxanthine, 137X), and 5-acetylamino-6-amino-3-methyluracil (AAMU).

#### Definition of CVD

Participants were identified as CVD patients if the answer was “yes” to any of the following question (22), “Has a doctor or other health professional ever told you that you have congestive heart failure (CHF)/coronary heart disease (CHD)/angina pectoris/heart attack/stroke?”

#### Definition of eGFR

eGFR was calculated by the Chronic Kidney Disease Epidemiology Collaboration (CKD-EPI) (23): = 141 × min (Scr/κ,1) α × max (Scr/κ, 1) −1.029 × 0.993 age × 1.108 (if female) × 1.159 (if black), κ is 0.7 for females and 0.9 for males, α is −0.329 for females and −0.411 for males, min indicates the minimum of Scr/κ or 1, and max indicates the maximum of Scr/κ or 1. Scr stands for serum creatinine (mg/dL).

### Outcome Variables and Follow-Up

Outcomes were recorded. CVD death was defined as a death caused by CHF, CHD, angina pectoris, heart attack or stroke. All-cause death was defined as all deaths. The end time of follow-up was 2015 with a median follow-up time of 113.5 (63, 133) months. The follow-up was terminated once patients occurred death in this study.

### Statistical Analysis

In the present study, the data of normally distributed was represented by mean ± standard deviation (Mean ± SD), and intergroup comparison adopted *t*-test. The data of non-normally distributed were described by the median with interquartile spacing [M (Q1, Q3)], Mann-Whitney U rank sum test was used to perform the comparison between groups. The number of cases and composition ratio *N* (%) was used to express the categorical variables, Chi-square or Fisher's exact test was applied for intergroup comparison.

We employed the Cox model to explore the relationship of coffee, tea, caffeine, decaffeinated coffee/tea intake, and urinary caffeine and caffeine metabolite on the risk of all-cause death in CVD population. Considering that the competitive-risk model could better reflect the real death risk of related to CVD, we established a competitive-risk model to investigate the association between coffee, tea, caffeine, decaffeinated coffee/tea intake and the risk of CVD specific-death in CVD population. In the current study, relevant confounders were adjusted in three models: Model 1 was unadjusted, Model 2 adjusted for age and BMI. Model 3 adjusted age, BMI, cancer, eGFR, energy, the history of CVD medications, carbohydrate and family PIR. Hazard ratios (HR) and 95% confidence interval (CI) were computed in the study. The missing values were interpolated by SAS (Supplementary Table 1). All statistical analyses were performed by SAS and Python, and *P* < 0.05 was regarded as statistically significant for all tests.

## Results

### Baseline Characteristics

After excluding some patients who were not CVD (*n* = 19,221), had incomplete information of coffee and tea consumption (*n* = 344), and had extreme total energy intakes (*n* = 279), a total of 626 eligible patients were enrolled in the study ultimately. These patients were divided into survival group (*n* = 304), non-CVD death group (*n* = 223), and CVD death group (*n* = 99). The incidence of all-cause death and CVD death was ~51.44 and 15.81% in the study. As shown in Table 1, the mean age was 66.85 years and the mean BMI was 29.73 kg/m^2^ in this population. In addition, most of CVD patients (64.04%) had coffee intake with 1–3 cups per day, 84.43% CVD patients had iced tea intake with <1 cup per day, and 90.71% CVD patients had hot tea intake with <1 cup per day. Detailed baseline information was given in Table 1.

**Table 1:** Baseline characteristics of all included participants.

**Variables**	**Total (*n =* 626)**	**Survival group (*n =* 304)**	**Non-CVD death group (*n =* 223)**	**CVD death group (*n =* 99)**	***P***
Gender, *n* (%)					0.312
Male	358 (53.07)	157 (49.95)	134 (57.02)	67 (56.75)	
Female	268 (46.93)	147 (50.05)	89 (42.98)	32 (43.25)	
Age, year, Mean (S.E)	66.85 (0.81)	62.18 (0.94)	72.24 (1.03)	73.59 (1.48)	<0.001
BMI, kg/m^2^, Mean (S.E)	29.73 (0.31)	30.22 (0.47)	28.93 (0.39)	29.57 (1.03)	0.101
Race, *n* (%)					0.340
Mexican American	78 (3.07)	43 (3.60)	25 (2.44)	10 (2.31)	
Other Hispanic	6 (0.74)	4 (0.82)	1 (0.47)	1 (1.07)	
Non-Hispanic White	426 (83.64)	179 (80.91)	171 (86.42)	76 (88.43)	
Non-Hispanic Black	96 (8.43)	69 (11.08)	18 (5.26)	9 (4.92)	
Other Race	20 (4.12)	9 (3.60)	8 (5.42)	3 (3.26)	
Education, *n* (%)					0.072
At least high school	415 (74.57)	209 (78.64)	140 (68.91)	66 (70.89)	
Less than high school	210 (25.43)	95 (21.36)	82 (31.09)	33 (29.11)	
Family income to poverty ratio (PIR), Mean (S.E)	2.72 (0.08)	2.87 (0.12)	2.46 (0.11)	2.69 (0.17)	0.040
Smoking status, *n* (%)					0.794
Yes	330 (53.54)	166 (54.74)	113 (52.59)	51 (50.80)	
No	296 (46.46)	138 (45.26)	110 (47.41)	48 (49.20)	
Alcohol, gm, Mean (S.E)	6.89 (1.03)	7.49 (1.58)	5.68 (1.36)	7.21 (2.10)	0.634
Protein, gm, Mean (S.E)	71.83 (1.86)	73.70 (2.53)	71.42 (3.31)	65.10 (3.29)	0.123
Cholesterol, *n* (%)					0.111
Yes	580 (93.31)	277 (91.71)	207 (94.25)	96 (97.67)	
No	46 (6.69)	27 (8.29)	16 (5.75)	3 (2.33)	
Total fat, gm, Mean (S.E)	72.53 (2.05)	75.17 (3.27)	71.60 (3.78)	63.88 (4.25)	0.072
Carbohydrate, gm, Mean (S.E)	227.91 (6.03)	236.20 (7.52)	224.39 (9.21)	202.02 (9.36)	0.008
eGFR, mL/min/m^2^, Mean (S.E)	68.34 (0.94)	74.85 (1.34)	60.35 (1.87)	60.08 (2.64)	<0.001
Energy, 10 kcal, Mean (S.E)	183.93 (4.01)	188.62 (5.55)	182.28 (6.22)	168.53 (6.85)	0.015
Hypertension, *n* (%)					0.340
Yes	446 (70.69)	217 (68.40)	68 (73.48)	161 (73.78)	
No	180 (29.31)	87 (31.60)	31 (26.52)	61 (26.22)	
The history of CVD medications, *n* (%)					<0.001
Yes	525 (82.07)	235 (75.30)	201 (89.79)	89 (92.05)	
No	101 (17.93)	69 (24.70)	22 (10.21)	10 (7.95)	
Diabetes					0.223
Yes	11 (1.59)	7 (2.12)	2 (0.81)	2 (1.20)	
No	174 (31.81)	99 (35.72)	48 (26.29)	27 (28.50)	
Unknown	441 (66.60)	198 (62.16)	173 (72.90)	70 (70.30)	
Cancer, *n* (%)					<0.001
Yes	114 (19.07)	41 (13.91)	51 (26.67)	22 (22.71)	
No	512 (80.93)	263 (86.09)	172 (73.33)	77 (77.29)	
Coffee, *n* (%)					0.191
<1 cup per day	137 (21.80)	75 (25.48)	43 (17.66)	19 (16.23)	
1–3 cups per day	412 (64.04)	192 (60.51)	153 (68.98)	67 (67.12)	
≥4 cups per day	77 (14.16)	37 (14.01)	27 (13.36)	13 (16.66)	
Caffeine, 100 mg, Mean (S.E)	2.05 (0.11)	2.03 (0.18)	2.14 (0.19)	1.91 (0.22)	0.794
Iced tea, *n* (%)					0.773
<1 cup per day	531 (84.43)	264 (86.23)	183 (81.14)	84 (84.65)	
1–3 cups per day	78 (11.47)	34 (9.89)	31 (14.38)	13 (11.26)	
≥4 cups per day	17 (4.09)	6 (3.88)	9 (4.48)	2 (4.09)	
Hot tea, *n* (%)					0.773
<1 cup per day	570 (90.71)	279 (90.28)	207 (92.81)	84 (87.68)	
1–3 cups per day	49 (7.54)	21 (7.62)	13 (5.33)	15 (12.32)	
≥4 cups per day	7 (1.74)	4 (2.10)	3 (1.86)	0 (0.00)	
Decaffeinated coffee, *n* (%)					0.547
Almost never	335 (55.33)	153 (52.85)	127 (59.32)	55 (56.30)	
About a quarter to three quarters of the time	101 (16.16)	58 (18.31)	30 (14.03)	13 (12.28)	
Always	190 (28.51)	93 (28.84)	66 (26.65)	31 (31.42)	
Decaffeinated iced tea, *n* (%)					<0.001
Almost never	447 (71.75)	199 (65.04)	177 (82.95)	71 (73.50)	
About a quarter to three quarters of the time	103 (16.63)	68 (22.14)	24 (9.40)	11 (10.69)	
Always	76 (11.62)	37 (12.82)	22 (7.65)	17 (15.81)	
Decaffeinated hot tea, *n* (%)					<0.001
Almost never	447 (72.22)	204 (67.73)	178 (82.49)	65 (66.93)	
About a quarter to three quarters of the time	85 (13.75)	56 (17.75)	17 (6.97)	12 (12.97)	
Always	94 (14.04)	44 (14.53)	28 (10.54)	22 (20.10)	
Follow-up time, months, Mean (S.E)	104.07 (2.56)	131.71 (2.01)	70.70 (2.64)	67.64 (5.06)	<0.001

*BMI, body mass index; CVD, cardiovascular disease; eGFR, estimated glomerular filtration rate*.

### The Associated Factors With the Risk of the All-Cause Death in CVD Population

**Figure 1:**
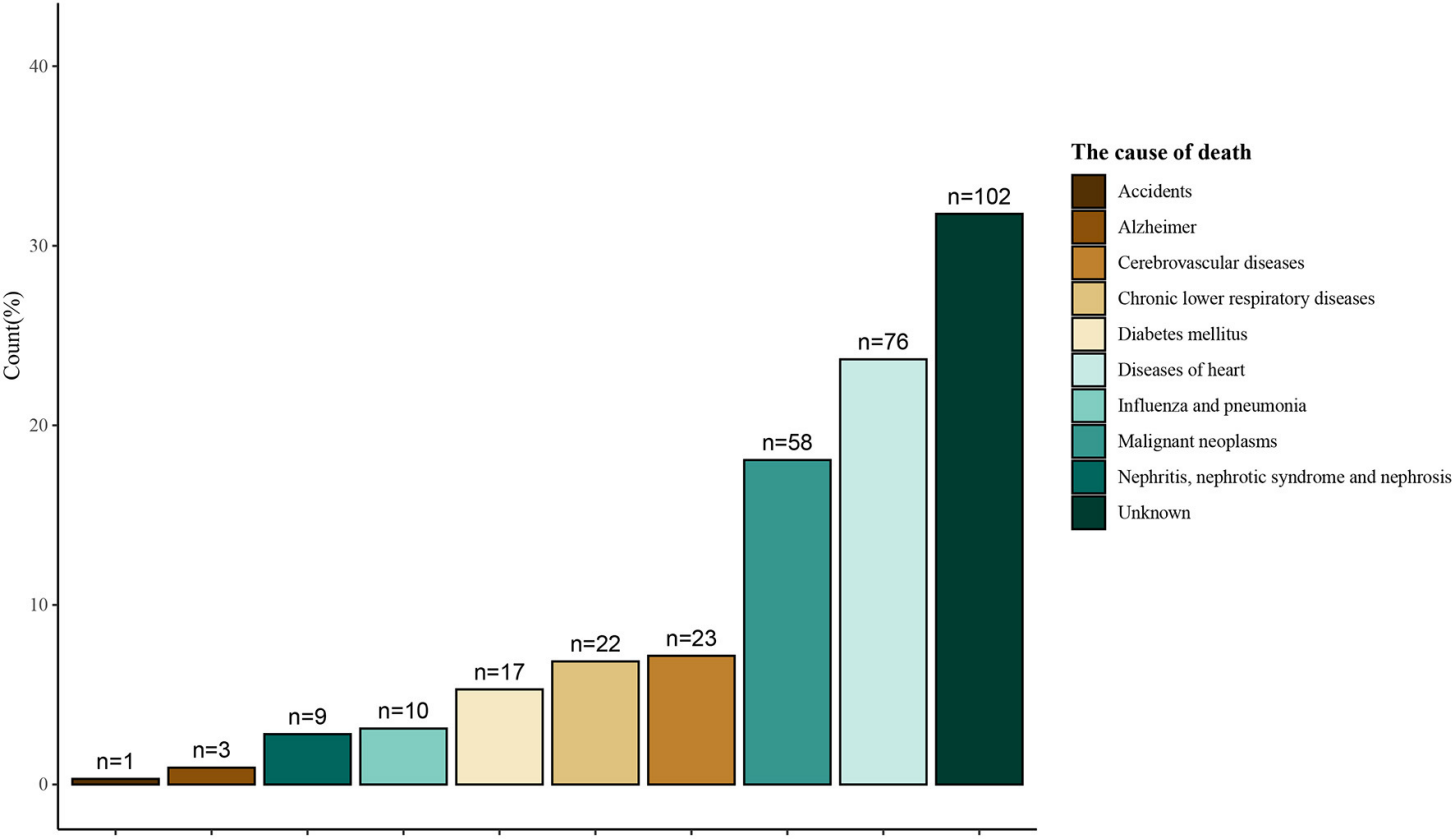
The causes of the all-cause death of CVD patients.

Table 2 shows the relationship of coffee consumption, caffeine consumption, iced tea consumption, hot tea consumption, and decaffeinated coffee/iced tea/hot tea on the risk of the all-cause death among CVD population. After adjusting for relevant confounders (*P* < 0.05), the results displayed that coffee consumption, caffeine consumption, iced tea consumption, hot tea consumption (≥4 cups per day) were associated with an increased risk of the all-cause death in CVD patients (Model 3). Nevertheless, hot tea consumption (1–3 cups per day), decaffeinated coffee, decaffeinated iced tea, and decaffeinated hot tea might reduce the risk of the all-cause death among CVD population. Additionally, Figure 1 also reveals the causes death of CVD patients.

**Table 2:** The related factors with the risk of the all-cause death in CVD population.

**Variables**	**Model 1**	***P***	**Model 2**	***P***	**Model 3**	***P***
	**HR (95%CI)**		**HR (95%CI)**		**HR (95%CI)**	
**Coffee**
<1 cup per day	Ref		Ref		Ref	
1–3 cups per day	1.425 (1.422–1.428)	<0.001	1.064 (1.062–1.066)	<0.001	1.093 (1.091–1.095)	<0.001
≥4 cups per day	1.261 (1.258–1.264)	<0.001	1.271 (1.268–1.274)	<0.001	1.389 (1.385–1.393)	<0.001
Caffeine	0.982 (0.982–0.983)	<0.001	1.103 (1.102–1.103)	<0.001	1.106 (1.105–1.106)	<0.001
**Iced tea**						
<1 cup per day	Ref		Ref		Ref	
1–3 cups per day	1.248 (1.246–1.251)	<0.001	1.211 (1.208–1.213)	<0.001	1.279 (1.276–1.282)	<0.001
≥4 cups per day	1.086 (1.083–1.090)	<0.001	1.795 (1.789–1.801)	<0.001	1.639 (1.632–1.645)	<0.001
**Hot tea**						
<1 cup per day	Ref		Ref		Ref	
1–3 cups per day	1.054 (1.052–1.057)	<0.001	0.838 (0.836–0.840)	<0.001	0.944 (0.941-0.946)	<0.001
≥4 cups per day	1.011 (1.005–1.017)	<0.001	1.543 (1.534–1.552)	<0.001	1.529 (1.520–1.538)	<0.001
**Decaffeinated coffee**						
Almost never	Ref		Ref		Ref	
About a quarter to three quarters of the time	0.761 (0.760–0.763)	<0.001	0.841 (0.839–0.842)	<0.001	0.869 (0.867–0.871)	<0.001
Always	0.909 (0.907–0.910)	<0.001	0.765 (0.764–0.767)	<0.001	0.787 (0.786–0.789)	<0.001
**Decaffeinated iced tea**						
Almost never	Ref		Ref		Ref	
About a quarter to three quarters of the time	0.422 (0.421–0.423)	<0.001	0.644 (0.634–0.646)	<0.001	0.697 (0.695–0.698)	<0.001
Always	0.760 (0.758–0.761)	<0.001	0.774 (0.773–0.776)	<0.001	0.772 (0.771–0.774)	<0.001
**Decaffeinated hot tea**						
Almost never	Ref		Ref		Ref	
About a quarter to three quarters of the time	0.503 (0.502–0.504)	<0.001	0.600 (0.598–0.601)	<0.001	0.611 (0.609–0.612)	<0.001
Always	0.934 (0.932–0.936)	<0.001	0.905 (0.903–0.906)	<0.001	0.943 (0.941–0.945)	<0.001

*HR, hazard ratios; CI, confidence interval*.

*Model 1: unadjusted*.

*Model 2: adjusted for age and BMI*.

*Model 3: adjusted for age, BMI, cancer, eGFR, energy, the history of CVD medications, carbohydrate and family PIR*.

Also, Table 3 shows the estimated association of urinary caffeine and caffeine metabolites and the risk of the all-cause death in CVD population by using Cox model. After adjusting for age, BMI, cancer, eGFR, energy, the history of CVD medications, carbohydrate and family PIR (Model 3), 1X (HR = 1.003, 95%CI: 1.000–1.006) was significantly associated with higher risk of all-cause death in CVD population.

**Table 3:** The association of urinary caffeine metabolites and the risk of the all-cause death in CVD population.

**Variables**	**Model 1**	***P***	**Model 2**	***P***	**Model 3**	***P***
	**HR (95%CI)**		**HR (95%CI)**		**HR (95%CI)**	
1-methyluric acid, umol/L	1.002 (1.000–1.003)	0.006	1.002 (1.000–1.003)	0.004	1.001 (1.000–1.003)	0.142
3-methyluric acid, umol/L	1.052 (1.019–1.084)	0.002	1.055 (1.017–1.093)	0.006	1.037 (0.982–1.091)	0.198
7-methyluric acid, umol/L	1.002 (0.999–1.004)	0.139	1.002 (1.000–1.005)	0.097	1.002 (0.996–1.007)	0.551
1,3-dimethyluric acid, umol/L	1.004 (0.999–1.009)	0.095	1.004 (0.999–1.008)	0.133	1.003 (0.996–1.011)	0.383
1,7-dimethyluric acid, umol/L	1.003 (1.000–1.005)	0.032	1.002 (1.000–1.004)	0.055	1.002 (0.998–1.005)	0.310
3,7-dimethyluric acid, umol/L	1.016 (0.952–1.080)	0.624	1.029 (0.971–1.087)	0.334	1.033 (0.959–1.107)	0.391
1,3,7-trimethyluric acid, umol/L	1.015 (0.985–1.045)	0.334	1.014 (0.987–1.042)	0.314	1.017 (0.978–1.057)	0.398
1-methylxanthine, umol/L	1.002 (0.999–1.005)	0.188	1.003 (1.000–1.006)	0.020	1.005 (1.000–1.009)	0.035
3-methylxanthine, umol/L	1.002 (1.000–1.004)	0.059	1.002 (1.000–1.004)	0.079	1.001 (0.997–1.005)	0.662
7-methylxanthine, umol/L	1.001 (0.999–1.002)	0.323	1.001 (1.000–1.003)	0.150	1.001 (0.999–1.004)	0.274
1,3-dimethylxanthine (theophylline), umol/L	0.978 (0.900–1.057)	0.586	0.994 (0.927–1.061)	0.865	1.007 (0.926–1.087)	0.869
1,7-dimethylxanthine (paraxanthine), umol/L	0.997 (0.987–1.007)	0.589	1.003 (0.993–1.014)	0.529	1.007 (0.990–1.025)	0.414
3,7-dimethylxanthine (theobromine), umol/L	0.999 (0.992–1.006)	0.698	0.999 (0.992–1.005)	0.719	0.995 (0.980–1.010)	0.524
1,3,7-trimethylxanthine (caffeine), umol/L	1.010 (0.993–1.027)	0.261	1.010 (0.994–1.027)	0.233	1.014 (0.990–1.039)	0.243
AAMU, umol/L	1.001 (1.000–1.002)	0.020	1.002 (1.000–1.003)	0.005	1.001 (1.000–1.003)	0.095

*HR, hazard ratios; CI, confidence interval*.

*Model 1: unadjusted*.

*Model 2: adjusted for age and BMI*.

*Model 3: adjusted for age, BMI, cancer, eGFR, energy, the history of CVD medications, carbohydrate and family PIR*.

### The Associated Factors With Risk of the CVD Death in CVD Population

Likewise, we also assessed the association between coffee consumption, caffeine consumption, iced tea consumption, hot tea consumption, decaffeinated coffee/iced tea/hot tea and the risk of the CVD death among CVD patients. As shown in Table 4, the results of Model 3 described that coffee consumption, caffeine consumption, iced tea consumption (≥4 cups per day, HR = 1.773, 95%CI: 1.763–1.784), hot tea consumption, decaffeinated iced tea (always, HR = 1.434, 95%CI: 1.430–1.439), decaffeinated hot tea (HR = 1.725, 95%CI: 1.719–1.731) could enhance the risk of the CVD death in CVD population during the follow-up period. Conversely, iced tea consumption (1–3 cups per day, HR = 0.870, 95%CI: 0.866–0.873), decaffeinated coffee, decaffeinated iced tea (about a quarter to three quarters of the time, HR = 0.728, 95%CI: 0.725–0.730) were related to the decreased risk of the CVD death in CVD patients.

**Table 4:** The related factors with the risk of the CVD death in CVD population.

**Variables**	**Model 1**	***P***	**Model 2**	***P***	**Model 3**	***P***
	**HR (95%CI)**		**HR (95%CI)**		**HR (95%CI)**	
**Coffee**
<1 cup per day	Ref		Ref		Ref	
1–3 cups per day	1.398 (1.394–1.402)	<0.001	1.125 (1.122–1.129)	<0.001	1.266 (1.262–1.271)	<0.001
≥4 cups per day	1.545 (1.539–1.551)	<0.001	1.483 (1.477–1.489)	<0.001	2.027 (2.018–2.036)	<0.001
Caffeine	0.949 (0.948–0.949)	<0.001	1.082 (1.081–1.083)	<0.001	1.126 (1.125–1.127)	<0.001
**Iced tea**						
<1 cup per day	Ref		Ref		Ref	
1–3 cups per day	0.936 (0.932–0.939)	<0.001	0.863 (0.859–0.866)	<0.001	0.870 (0.866–0.873)	<0.001
≥4 cups per day	0.902 (0.898–0.907)	<0.001	1.717 (1.707–1.727)	<0.001	1.773 (1.763–1.784)	<0.001
**Hot tea**						
<1 cup per day	Ref		Ref		Ref	
1–3 cups per day	2.318 (2.310–2.326)	<0.001	1.911 (1.905–1.918)	<0.001	2.005 (1.998–2.012)	<0.001
≥4 cups per day^*^	–	<0.001	–	<0.001	–	<0.001
**Decaffeinated coffee**						
Almost never	Ref		Ref		Ref	
About a quarter to three quarters of the time	0.772 (0.769–0.775)	<0.001	0.826 (0.822–0.829)	<0.001	0.901 (0.897–0.904)	<0.001
Always	0.982 (0.979–0.984)	<0.001	0.777 (0.775–0.780)	<0.001	0.724 (0.722–0.726)	<0.001
**Decaffeinated iced tea**						
Almost never	Ref		Ref		Ref	
About a quarter to three quarters of the time	0.588 (0.586–0.591)	<0.001	0.708 (0.705–0.710)	<0.001	0.728 (0.725–0.730)	<0.001
Always	1.397 (1.392–1.401)	<0.001	1.497 (1.492–1.502)	<0.001	1.434 (1.430–1.439)	<0.001
**Decaffeinated hot tea**						
Almost never	Ref		Ref		Ref	
About a quarter to three quarters of the time	0.892 (0.889–0.895)	<0.001	1.081 (1.077–1.084)	<0.001	0.955 (0.952–0.959)	<0.001
Always	1.957 (1.950–1.963)	<0.001	1.859 (1.853–1.865)	<0.001	1.725 (1.719–1.731)	<0.001

*HR, hazard ratios; CI, confidence interval*.

*Model 1: unadjusted*.

*Model 2: adjusted for age and BMI*.

*Model 3: adjusted for age, BMI, cancer, eGFR, energy, the history of CVD medications, carbohydrate and family PIR*.

The symbol ^*^ indicates that the sample size was 0 and no calculation was performed.

## Discussion

In this retrospective cohort study of 626 patients were diagnosed with CVD, we observed that the high consumption of coffee, tea, and caffeine was associated with an increased risk of all-cause and specific-death for CVD patients. Our study also found that decaffeinated coffee/tea could decrease the all-cause death risk in CVD patients. Decaffeinated coffee could affect the lower risk of CVD death with respect to the CVD patients. Additionally, we also found that urinary caffeine metabolites level (1X) showed a significant positive association on the risk of all-cause death in CVD population.

In the present study, the findings showed that higher consumption of coffee, tea and caffeine enhanced the risk of all-cause and CVD death for CVD patients, which suggested that CVD patients should drink moderate amount of coffee, tea and as well as caffeine intake. Caffeine is a methylxanthine alkaloids and stimulant alkaloid of central nervous system, and commonly found in the coffee, tea, soft drinks, and chocolate (24). Currently, caffeine is thought to affect the cardiovascular system mainly through anti-adenosine receptors, inhibiting phosphodiesterase activity, promoting the release of catecholamines from adrenal glands, activating sympathetic nerves and renin-angiotensin system (25, 26). Our findings showed the fact that drinking 1–3 cups of iced tea per day was associated with a reduced risk of CVD death for CVD patients, the possible reason may be related to the solubility of active components. It is well-known that the solubility of caffeine decreases with increasing temperature (27), which also suggested that caffeine might have a less solubility and bioavailability in iced tea. We speculated when patients with CVD drank moderate iced tea, the bioavailability of caffeine reduce, which may decrease the risk of hyperactive sympathetic system by caffeine and be beneficial to the health of CVD patients (28, 29). However, interestingly, more than 4 cups of iced tea per day was considered as a risk factor in the CVD specific-death, this may be related to temperature or population selection, more research needs to be explored in detail. Notedly, among urinary caffeine metabolites, 1X was related to an increased risk of death for CVD patients. Hence, our findings also indicated that 1X exerts a more pronounced effect than other metabolites, leading to a rise in the likelihood of death of CVD patients.

Torres-Collado et al. pointed out that there was not significant association between the decaffeinated coffee and risk of CVD death in a representative sample of an adult population in Valencia and Spain (30), which was inconsistent with our results. In our study, decaffeinated coffee/tea was significantly related to the decreased risk for the CVD all-cause death, which indicated that decaffeinated coffee/tea might play a protective effect for death for CVD patients. Coffee and tea contain a variety of other chemicals besides caffeine. For instance, coffee contains polyphenols, such as chlorogenic acids, alkaloids, phenolic compounds, trace elements and so on (31); tea is also rich in tea polyphenols, catechin polymers, tea polysaccharides and other functional components as well as other natural products (32). Chlorogenic acids, alkaloids, phenolic compounds, trace elements, tea polyphenols, catechin polymers and tea polysaccharides have been proved to play vital roles in the anti-oxidant, anti-inflammatory, and anti-thrombosis, improving endothelial function, inhibiting the aggregation of platelet, reducing blood sugar and blood lipid, regulating metabolism and improving intestinal microbiome (32–34). This might be the underlying mechanism that decaffeinated coffee/tea decreased the death risk for CVD patients.

With respect to the coffee and tea, this study showed that higher coffee and tea consumption increased the death risk for CVD patients, and while decaffeinated coffee/tea was considered to play a protective effect for death for CVD patients. Some studies have shown that coffee and caffeine may increase the sympathetic nervous system of coffee drinkers, leading to autonomic imbalances (28, 29); for CVD patients, autonomic imbalance might cause the poor prognosis (29, 35). The findings suggest that caffeinated beverages should be consumed in moderation for patients with CVD. More studies are required to further confirm this association and give more evidences about mechanism.

The current study has several limitations. Firstly, the study had a relatively small sample size, which might have limited the statistical power, but the follow-up period of the study was long enough to investigate the association between coffee, tea, caffeine, decaffeinated coffee/tea consumption and risk of all-cause death and CVD death in CVD population. Secondly, laboratory measures such as caffeine, were measured only twice in 2 days and are not necessarily representative. Lastly, because all information in this retrospective cohort study was derived from the NHANES database, we did not collect the information about the consumption of medications prescribed for patients with CVD, the types of coffee or tea consumption, duration of brewing, the method of coffee or tea preparation and beverage cup sizes, more researches are needed to explore this association.

## Conclusion

In conclusion, our study indicated that for CVD patients, higher consumption of coffee, tea and caffeine could increase the risk of all-cause and CVD death. More studies are needed to further elucidate the association.

## Data Availability Statement

Publicly available datasets were analyzed in this study. This data can be found here: https://www.cdc.gov/nchs/nhanes/index.htm.

## Ethics Statement

Ethical approval was not provided for this study on human participants because due to the data from NHANES database were publicly available, this study did not require institutional review board approval. The patients/participants provided their written informed consent to participate in this study.

## Author Contributions

HZ, FL, and PZ designed the study and wrote the manuscript. HZ, FL, and NX collected and analyzed the data. HZ and LY contributed to literature search. PZ and FL critically reviewed and improved the drafts of the manuscript. All authors have read and approved the final manuscript.

## Funding

This research was supported by Major project of Fujian Science and Technology Program (2016YZ0001-1) and Excellent Young Doctor Training Program in Fujian Provincial Health System (2013-ZQN-ZD-4).

## Conflict of Interest

The authors declare that the research was conducted in the absence of any commercial or financial relationships that could be construed as a potential conflict of interest.

## Publisher's Note

All claims expressed in this article are solely those of the authors and do not necessarily represent those of their affiliated organizations, or those of the publisher, the editors and the reviewers. Any product that may be evaluated in this article, or claim that may be made by its manufacturer, is not guaranteed or endorsed by the publisher.
