# Supplementary material for: Association of Coffee, Tea, and Caffeine Consumption With All-Cause Risk and Specific Mortality for Cardiovascular Disease Patients
Source: Front Nutr. 2022 Jun 23;9:842856. doi: 10.3389/fnut.2022.842856 (PMC9261910; doi:10.3389/fnut.2022.842856)
Supplement: Supplementary file 1 [file Table_1.DOCX]

**Supplemental Table 1** The sensitivity analysis of missing data before and after interpolation

| Variables | Ratio of missing values (%) | Before the interpolation | After the interpolation | Statistics | *P* |
| --- | --- | --- | --- | --- | --- |
| BMI, Mean (S.E) | 72 (1.49) | 28.20 (0.18) | 28.06 (0.18) | t=1.80 | 0.082 |

**Note:** BMI: body mass index.
